# Supplementary material for: Attitudes of Patients with Adrenoleukodystrophy towards Sex-Specific Newborn Screening
Source: Int J Neonatal Screen. 2023 Sep 2;9(3):51. doi: 10.3390/ijns9030051 (PMC10531683; doi:10.3390/ijns9030051)
Supplement: Supplementary file 1 [file IJNS-09-00051-s001.zip › Supplementary File S1 Questionnaire.pdf]

Supplementary file S1: Questionnaire ALD and newborn screening

Vragenlijst: ALD en de hielprik

1. Uw gegevens

1. U bent:
a. Man
b. Vrouw
c. Overig

2. Wat is uw leeftijd?
a. ……. jaar

Uw ervaring met ALD

1. Wie in uw gezin of familie heeft of hebben ALD? (meerdere antwoorden mogelijk)
a. Ikzelf
b. Mijn partner
c. Mijn zoon(s), aantal …
d. Mijn dochter(s), aantal …
e. Mijn vader
f. Mijn moeder
g. Mijn broer(s), aantal …
h. Mijn zus(sen), aantal …
i. Mijn kleinzoon(s), aantal …
j. Mijn kleindochter(s), aantal …
k. Anders, namelijk …

Beantwoord de vragen 2 t/m 4 alleen als u zelf ALD heeft.

2. Op welke leeftijd is de diagnose ALD bij u gesteld?
a. Kinderleeftijd (t/m 18 jaar)
b. Volwassen leeftijd
c. Weet ik niet

3. Hoe is de diagnose ALD bij u gesteld? Vanwege:
a. Klachten door ALD
b. Een familielid met ALD
c. Een onderzoek tijdens de zwangerschap (prenatale diagnostiek)
d. Anders, namelijk …
e. Weet ik niet

4. Welke klachten van ALD heeft u (gehad)? (meerdere antwoorden mogelijk)
a. Geen, ik ben altijd klachtenvrij geweest

b. Ruggenmergschade (myelopathie; met bijvoorbeeld loop- of plasproblemen tot gevolg)
c. Bijnierschors problemen (bijnierschorsinsufficiëntie; de ziekte van Addison)
d. Een hersenbeschadiging (cerebrale ALD; zichtbaar op een MRI van het hoofd)
e. Anders, namelijk …
f. Weet ik niet

Beantwoord vraag 5 alleen als u klachten van ALD heeft (gehad).

5. Hoe lang was de periode tussen uw eerste klachten en het moment dat ALD bij u werd vastgesteld?
a. … maanden/jaar

Beantwoord vraag 6 t/m 8 alleen als u een familielid heeft of had met ALD.

6. Op welke leeftijd is de diagnose ALD bij uw familielid/familieleden gesteld?
a. Kinderleeftijd (t/m 18 jaar)
b. Volwassen leeftijd
c. De diagnose is bij verschillende familieleden op verschillende leeftijden vastgesteld (zowel op de kinderleeftijd als op de volwassen leeftijd)
d. Weet ik niet

7. Hoe is de diagnose bij uw familielid/familieleden gesteld? Vanwege (meerdere antwoorden mogelijk):
a. Klachten door ALD
b. Een ander familielid met ALD
c. De hielprik
d. Een onderzoek tijdens de zwangerschap (prenatale diagnostiek)
e. Anders, namelijk …
f. Weet ik niet

8. Welke klachten van ALD heeft uw familielid/hebben uw familieleden (gehad)? (meerdere antwoorden mogelijk)
a. Geen, hij/zij is/zijn altijd klachtenvrij geweest
b. Ruggenmergschade (myelopathie; met bijvoorbeeld loop- of plasproblemen tot gevolg)
c. Bijnierschors problemen (bijnierschorsinsufficiëntie; de ziekte van Addison)
d. Een hersenbeschadiging (cerebrale ALD; zichtbaar op een MRI van het hoofd)
e. Anders, namelijk …
f. Weet ik niet

9. Heeft u een familielid of meerdere familieleden met ALD die is/zijn overleden aan ALD?
a. Ja, 1 familielid/meerdere familieleden, namelijk (bijv. kind(eren), broer, zus): ……….
b. Nee

2. De hielprik bij baby's

De volgende vragen gaan over uw mening over de hielprik. In de eerste week na de geboorte van een baby worden een paar druppels bloed afgenomen uit de hiel van het kind. Het laboratorium onderzoekt het bloed op een aantal ernstige, zeldzame aangeboren ziektes. Dit zijn ziektes die niet veel baby's krijgen, maar die wel ernstig zijn. Als deze ziektes vroeg worden ontdekt, kan het kind beter behandeld worden. De gevolgen van de ziekte zijn dan minder erg. De Nederlandse overheid betaalt en regelt de hielprik.

1. Kruis aan: de hielprik bij baby's vind ik:

	1	2	3	4	5	
Slecht						Goed
Nutteloos						Nuttig
Niet betrouwbaar						Betrouwbaar

2. Heeft of hebben uw kinderen de hielprik gehad?

a. Ja, al mijn kinderen
b. Een deel van mijn kinderen
c. Geen van mijn kinderen
d. Ik heb geen kinderen
e. Weet ik niet
f. Anders namelijk …

Beantwoordt de volgende vraag alleen als u één of meerdere kinderen heeft:

3. Indien u niet meegedaan heeft met de hielprik, wat was hiervoor de reden?

a. Ik heb wel meegedaan met de hielprik
b. Er was nog geen hielprik
c. Anders, namelijk …

3. ALD in de hielprik.

ALD geeft bij jongens andere klachten dan bij meisjes. Bijvoorbeeld:

Bij jongens...:
- kunnen problemen in de hersenen en bijnieren ontstaan waardoor zij kunnen overlijden als de behandeling niet op tijd wordt gestart.
- kan de ziekte goed behandeld worden als het vroeg wordt ontdekt.

Bij meisjes...:
- kunnen later in het leven (bij 40-60 jaar) andere, vaak mildere problemen ontstaan, bijvoorbeeld bij het lopen.
- kan de ziekte niet behandeld worden.

1. Vindt u het een goed idee als de hielprik op ALD test?
   - ○ Ja, ik vind het een GOED idee om op ALD te testen, maar ALLEEN bij JONGENS, omdat: ………..
   - ○ Ja, ik vind het een GOED idee om op ALD te testen, BIJ JONGENS EN MEISJES, omdat: ………..
   - ○ Nee, ik vind het GEEN goed idee om op ALD te testen, omdat: ………..

2. Geef aan of u het eens of oneens bent met de onderstaande stellingen (a t/m s).
   - *Vroege opsporing* betekent dat de diagnose ALD vlak na de geboorte wordt gesteld. In dit geval gebeurt dat met de hielprik.

a. ALD zou bij *meisjes* niet vroeg moeten worden opgespoord, omdat er geen behandeling is voor hun klachten.

	1	2	3	4	5	
Helemaal mee oneens	○	○	○	○	○	Helemaal mee eens

b. ALD zou bij *meisjes* niet vroeg moeten worden opgespoord, omdat zij (bijna) nooit levensbedreigende klachten ontwikkelen.

	1	2	3	4	5	
Helemaal mee oneens	○	○	○	○	○	Helemaal mee eens

c. ALD zou bij *meisjes* niet vroeg moeten worden opgespoord, omdat het weinig toevoegt aan hun kwaliteit van leven.

	1	2	3	4	5	
Helemaal mee oneens	○	○	○	○	○	Helemaal mee eens

d. ALD zou bij *meisjes* niet vroeg moeten worden opgespoord, omdat het mentaal belastend is om te weten dat zij op latere leeftijd misschien onbehandelbare klachten gaan ontwikkelen.

	1	2	3	4	5	
Helemaal mee oneens	○	○	○	○	○	Helemaal mee eens

e. ALD kan nadelige financiële gevolgen hebben, zoals bij het afsluiten van een verzekering.

	1	2	3	4	5	
Helemaal mee oneens	○	○	○	○	○	Helemaal mee eens

f. Vroege opsporing van ALD ontneemt ouders de kans om te genieten van een (nog) gezonde baby.

	1	2	3	4	5	
Helemaal mee oneens	○	○	○	○	○	Helemaal mee eens

g. Vroege opsporing van ALD leidt tot een verminderde hechting tussen ouders en kind.

	1	2	3	4	5	
Helemaal mee oneens	○	○	○	○	○	Helemaal mee eens

h. Het vroeg opsporen van ALD met de hielprik bij *zowel jongens als meisjes* is te duur voor de samenleving.

	1	2	3	4	5	
Helemaal mee oneens	○	○	○	○	○	Helemaal mee eens

i. Je moet het leven nemen zoals het komt.

	1	2	3	4	5	
Helemaal mee oneens	○	○	○	○	○	Helemaal mee eens

j. Ieder kind met ALD heeft recht op een ‘open’ toekomst.

	1	2	3	4	5	
Helemaal mee oneens	○	○	○	○	○	Helemaal mee eens

k. Het is oneerlijk als *meisjes* niet worden getest op ALD, maar *jongens* wel.

	1	2	3	4	5	
Helemaal mee oneens	○	○	○	○	○	Helemaal mee eens

l. ALD zou bij *jongens* wel vroeg moeten worden opgespoord, omdat het een lange periode tussen de eerste klachten en de uiteindelijke diagnose kan voorkomen.

	1	2	3	4	5	
Helemaal mee oneens	○	○	○	○	○	Helemaal mee eens

m. ALD zou bij *meisjes* wel vroeg moeten worden opgespoord, omdat het een lange periode tussen de eerste klachten en de uiteindelijke diagnose kan voorkomen.

	1	2	3	4	5	
Helemaal mee oneens	○	○	○	○	○	Helemaal mee eens

n. ALD zou bij *jongens* wel vroeg moeten worden opgespoord, omdat zij bij de eerste klachten dan direct optimale begeleiding krijgen.

	1	2	3	4	5	
Helemaal mee oneens	○	○	○	○	○	Helemaal mee eens

o. ALD zou bij *meisjes* wel vroeg moeten worden opgespoord, omdat zij bij de eerste klachten dan direct optimale begeleiding krijgen.

	1	2	3	4	5	
Helemaal mee oneens	○	○	○	○	○	Helemaal mee eens

p. ALD zou bij *jongens* wel vroeg moeten worden opgespoord zodat ouders op tijd kunnen worden voorgelicht over mogelijkheden rondom verdere gezinsuitbreiding.

	1	2	3	4	5	
Helemaal mee oneens	○	○	○	○	○	Helemaal mee eens

q. ALD zou bij *meisjes* wel vroeg moeten worden opgespoord zodat ouders op tijd kunnen worden voorgelicht over mogelijkheden rondom verdere gezinsuitbreiding.

	1	2	3	4	5	
Helemaal mee oneens	○	○	○	○	○	Helemaal mee eens

r. ALD zou bij *jongens* wel vroeg moeten worden opgespoord, omdat op die manier andere familieleden met ALD ook kunnen worden opgespoord.

	1	2	3	4	5	
Helemaal mee oneens	○	○	○	○	○	Helemaal mee eens

s. ALD zou bij *meisjes* wel vroeg moeten worden opgespoord, omdat op die manier andere familieleden met ALD (waaronder jongens en mannen) ook kunnen worden opgespoord.

	1	2	3	4	5	
Helemaal mee oneens	○	○	○	○	○	Helemaal mee eens

4. De ernst van ALD.

Omcirkel alstublieft bij elke vraag het getal dat uw mening het beste weergeeft.

1. Hoeveel beïnvloedt ALD iemands leven?

Helemaal geen invloed	0	1	2	3	4	5	6	7	8	9	10	Zeer veel invloed

2. Hoe lang denkt u dat ALD zal duren?

Een zeer korte tijd	0	1	2	3	4	5	6	7	8	9	10	Het hele leven

3. Hoeveel controle vindt u dat iemand heeft over ALD?

Helemaal geen controle	0	1	2	3	4	5	6	7	8	9	10	Zeer veel controle

4. Hoeveel denkt u dat behandeling kan helpen bij ALD?

Helemaal niet	0	1	2	3	4	5	6	7	8	9	10	Zeer veel

5. Hoe sterk ervaart iemand klachten door ALD?

Helemaal geen klachten	0	1	2	3	4	5	6	7	8	9	10	Zeer veel klachten

6. Hoe bezorgd bent u over ALD?

Helemaal niet bezorgd	0	1	2	3	4	5	6	7	8	9	10	Zeer bezorgd

7. In welke mate vindt u dat u ALD begrijpt?

Helemaal geen begrip	0	1	2	3	4	5	6	7	8	9	10	Zeer veel begrip

8. Hoeveel invloed heeft ALD op iemands stemming? (Bijvoorbeeld: maakt ALD iemand boos, bang, van streek of somber?)

Helemaal geen invloed	0	1	2	3	4	5	6	7	8	9	10	Zeer veel invloed

5. Achtergrondgegevens.

1. Mijn opleiding is:
a. Basisonderwijs
b. Vmbo-b/k, mbo1
c. Vmbo-g/t, havo-onderbouw of vwo-onderbouw
d. Mbo2 en mbo3
e. Mbo4
f. Havo, vwo
g. Hbo, wo-bachelor
h. Hbo-master, wo-master, doctor
i. Anders, (vul in) ______

2. In welk land bent u geboren?
a. Nederland
b. Een ander land binnen Europa, namelijk …
c. Een ander land buiten Europa, namelijk …

3. In welk land is uw moeder geboren?
a. Nederland
b. Een ander land binnen Europa, namelijk …
c. Een ander land buiten Europa, namelijk …
d. Weet ik niet

4. In welk land is uw vader geboren?
a. Nederland
b. Een ander land binnen Europa, namelijk …
c. Een ander land buiten Europa, namelijk …
d. Weet ik niet

5. Doet u veel met uw geloof?
a. Ik ben niet gelovig
b. Ik doe niets met mijn geloof
c. Ik ben een beetje actief binnen mijn geloof
d. Ik ben actief binnen mijn geloof

6. Is het volgende belangrijk voor u?
a. Antroposofie
b. Homeopathie
c. Natuurgeneeskunde
d. Geen van allen
e. Anders, namelijk …

7. Als u een of meerdere kinderen heeft of in de toekomst verwacht te krijgen, heeft u gegeven /gaat u hen vaccinaties geven? (bijv. tegen difterie, kinkhoest, tetanus en polio)
a. Ja, alle vaccinaties
b. Ja, maar niet alle vaccinaties
c. Nee, geen vaccinaties
d. Nee ik heb/wil geen kinderen
e. Anders, namelijk …

8. Heeft u opmerkingen over deze vragenlijst of wilt u graag nog iets toevoegen?
a. Ja, namelijk: …

………
………
………
………
………
………

Hartelijk dank voor het invullen van deze vragenlijst, mogen wij u op een later punt benaderen met aanvullende vragen?

- Ja/nee
1. Indien 'ja', mijn e-mail adres is: ………..
2. Indien 'ja', mijn telefoonnummer is: ………..
