# Supplementary material for: Attitudes of Patients with Adrenoleukodystrophy towards Sex-Specific Newborn Screening
Source: Int J Neonatal Screen. 2023 Sep 2;9(3):51. doi: 10.3390/ijns9030051 (PMC10531683; doi:10.3390/ijns9030051)
Supplement: Supplementary file 1 [file IJNS-09-00051-s001.zip › Supplementary Table S1.pdf]

Supplementary table S1: Open-text responses indicating reasons for respondents' preferences for screening

In favor of only screening boys (n=13)	
Boys develop severe symptoms	7 (46%)
Girls develop no/ less severe symptoms	3 (23%)
ALD is treatable in boys	2 (15%)
Important to know in case of child wish	2 (15%)
Parents can take immediate action after diagnosis	1 (8%)
ALD is not treatable in girls	1 (8%)
In favor of screening boys and girls (n=48)	
Helps to provide an early diagnosis	13 (27%)
Facilitates (early) treatment and monitoring	12 (25%)
Helps with family planning	8 (17%)
ALD has an impact on both sexes	7 (15%)
Helps make future plans	6 (13%)
Miscellaneous reasons	5 (10%)
No discrimination between sexes	3 (6%)
Helps prevent suffering	2 (4%)
Research and better understanding of the disease	2 (4%)
Enables extended family screening for ALD	1 (2%)

Reasons why respondents indicated that they preferred the indicated type of screening are ordered from most common to least common. Some open-text answers included multiple reasons. Not all respondents explained their choice. ALD: adrenoleukodystrophy.
